# The valley Zeeman effect in inter- and intra-valley trions in monolayer WSe_2_

**DOI:** 10.1038/s41467-019-10228-7

**Published:** 2019-05-27

**Authors:** T. P. Lyons, S. Dufferwiel, M. Brooks, F. Withers, T. Taniguchi, K. Watanabe, K. S. Novoselov, G. Burkard, A. I. Tartakovskii

**Affiliations:** 1 Department of Physics and Astronomy, University of Sheffield, Sheffield, S3 7RH UK; 2 Department of Physics, University of Konstanz, D-78464 Konstanz, Germany; 3 School of Physics and Astronomy, University of Manchester, Manchester, M13 9PL UK; 4 Centre for Graphene Science, CEMPS, University of Exeter, Exeter, EX4 4QF UK; 5 National Institute for Materials Science, Tsukuba, Ibaraki, 305-0044 Japan

**Keywords:** Two-dimensional materials, Two-dimensional materials, Magneto-optics, Near-infrared spectroscopy

## Abstract

Monolayer transition metal dichalcogenides (TMDs) hold great promise for future information processing applications utilizing a combination of electron spin and valley pseudospin. This unique spin system has led to observation of the valley Zeeman effect in neutral and charged excitonic resonances under applied magnetic fields. However, reported values of the trion valley Zeeman splitting remain highly inconsistent across studies. Here, we utilize high quality hBN encapsulated monolayer WSe_2_ to enable simultaneous measurement of both intervalley and intravalley trion photoluminescence. We find the valley Zeeman splitting of each trion state to be describable only by a combination of three distinct g-factors, one arising from the exciton-like valley Zeeman effect, the other two, trion specific, g-factors associated with recoil of the excess electron. This complex picture goes significantly beyond the valley Zeeman effect reported for neutral excitons, and eliminates the ambiguity surrounding the magneto-optical response of trions in tungsten based TMD monolayers.

## Abstract (web summary)

The unique valley and spin texture of atomically thin transition metal dichalcogenides (TMDs) allows the observation of the valley Zeeman effect for neutral and charged excitons. Here, the authors unveil the underlying physics of the magneto-optical response and valley Zeeman splitting of trions in tungsten-based TMDs.

## Introduction

Over the past several years, optical investigations of monolayer transition metal dichalcogenides (TMDs) have generated significant scientific interest^1^. These layered semiconductors show remarkable properties when reduced to a single atomic layer, such as an indirect to direct band gap transition^2,3^, alongside a regime of coupled spin and valley physics^4–6^. Low temperature photoluminescence (PL) of monolayer TMDs, such as tungsten diselenide (WSe_2_), exhibits spectra dominated by excitonic emission in the near infra-red, where a range of biexcitonic and trionic complexes have been reported^7–15^.

The valley degree of freedom exhibited by monolayer WSe_2_ and other TMDs, which allows excitons to occupy degenerate but momentum-opposite states within the Brillouin zone, opens prospects for information encoding and processing exploiting the valley pseudospin^16^. In WSe_2_ carriers may occupy either the +K or −K valleys, where they are robust against intervalley scattering due to the large momentum transfer needed to cross the Brillouin zone, and the energy transfer required to overcome the large spin-orbit splitting at the conduction and valence band edges, which is opposite in the two valleys by time reversal symmetry^17^. A further property of monolayer TMDs is the locking of the polarization of optically bright transitions to the valley pseudospin: electron hole pairs in the +K (−K) valley are coupled to *σ*^+^ (*σ*^−^) polarized light. This allows optical generation and addressability of valley polarized excitons^6^, along with their more elaborate complexes such as charged excitons (trions)^18^ and exciton-polaritons^19^.

A recently observed consequence of the coupled spin and valley regime inherent to monolayer WSe_2_ is the valley Zeeman effect^20–22^. Here, an external magnetic field, *B*, normal to the monolayer lifts the degeneracy between valley polarized states, such that excitonic resonances in the +K and −K valleys will shift spectrally away from one another. It has been reported that this energy splitting depends on two different magnetic moments, an intracellular contribution, arising from the tungsten d-orbitals in the valence band, and an intercellular contribution from finite Berry curvature at the +K and −K points^20–22^. Direct optical measurement of the valley Zeeman splitting is possible thanks to the locking of light helicity to the valley pseudospin, and allows extraction of a valley Zeeman g-factor for a given spectral resonance. While the valley Zeeman splitting of the neutral exciton is fairly consistently reported to be *E*(*σ*^+^) − *E*(*σ*^−^) ≈ −4 *μ*_B_*B*^20–22^, where *μ*_B_ is the Bohr magneton, values reported for the negatively charged trion vary from −4 *μ*_B_*B* to −13 *μ*_B_*B*^21–24^, and are the subject of some speculation and ambiguity as to the cause of the reported variation.

**Fig. 1:**
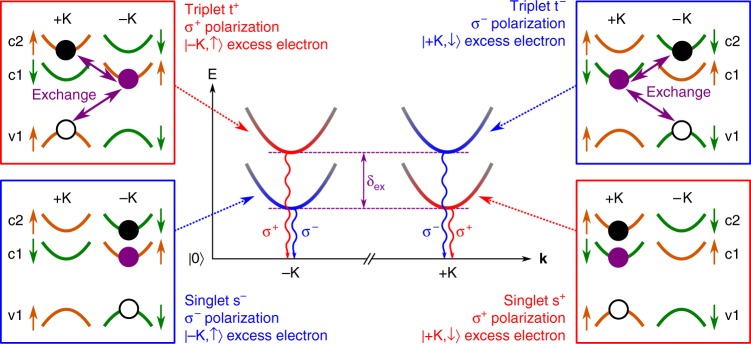
Scheme of singlet and triplet trions in WSe_2_. Generalized dispersion relations *E*(**k**), where **k** is the wavevector of the excess electron, of the four optically bright ground state negative trions in monolayer WSe_2_, following the convention established in refs. ^20,25,26^. Side panels illustrate the spin-valley configurations of the constituent carriers of each trion variety. v1 is the topmost spin-subband within the valence band, while c1 (c2) is the lower (upper) energy spin-orbit split conduction band. Based on optical selection rules, the black electron (black filled circle) and hole (black empty circle) are the recombining pair, while the purple electron (purple filled circle) is excess and occupies band c1. Orange (green) conduction and valence band states are spin up (down). Red (blue) colours denote *σ*^+^ (*σ*^−^) helicity of the bright transition. The trion states are labelled X^Y^ where X = s, t for singlet or triplet, Y = +, − for *σ*^+^ or *σ*^−^ emission helicity. Purple arrows indicate the intervalley Coulomb exchange interaction, present only in triplet trions, which raises the energy of the triplets by an amount *δ*_ex_ above the singlets. This energy gap gives rise to the trion fine structure in emission

In monolayer WSe_2_, optical selection rules dictate that negative trions must have an electron with the same spin and valley index as the hole in order to allow radiative recombination. As such, an electron must always occupy the upper spin state of the conduction band (c2 in Fig. 1), allowing the excess electron to occupy the lower energy conduction band spin state, in either valley (c1 in Fig. 1). This gives a total of four different ground state bright A-trion configurations, which are illustrated in Fig. 1. Two of these trion configurations are intravalley, with all three carriers in the same valley, and the other two are intervalley, with the excess electron in the opposite valley to the e-h pair which may recombine with the emission of a photon^18,20^. It is convenient to define these trion configurations as singlet and triplet trions, respectively, where the cumulative spin of the electron pair determines the classification. As a result we can define the four ground state trion configurations as s^+^, s^−^, t^+^, t^−^, where s and t denote singlet and triplet, and + and − denote the circular polarization of the optically bright transition of the state. For clarity these are labelled in Fig. 1.

The intervalley Coulomb exchange interaction between the e-h pair and the excess electron in triplet trions raises their energy relative to the singlet by an amount *δ*_ex_, expected to be a few meV^25^. In luminescence, this energy gap gives rise to trion fine structure^18,26^, as depicted schematically in Fig. 1 for the case of zero external magnetic field. In monolayer WS_2_, similar trion fine structure has been observed^27,28^, and magneto-optical measurements have uncovered inequivalent valley Zeeman g-factors for the two fine structure components^29^. However, no thorough explanation has been given for this difference. Furthermore, to our knowledge no detailed magneto-optical study of the WSe_2_ trion fine structure has yet been reported. It is highly likely that the complex nature of WSe_2_ trions, having four distinct valley configurations, is the root cause of the wide ranging and apparently random valley Zeeman g-factors so far measured.

In this work, we report that the relative intensity of singlet and triplet trions has a strong temperature dependence, such that heating a WSe_2_ monolayer from 4 to 30 K thermally populates the triplet states allowing simultaneous measurement of the magneto-optical response of the trion fine structure components. We observe that different trion valley configurations have inequivalent rates of field-dependent spectral shift, which is incompatible with the valley Zeeman interpretation reported for neutral excitons. We extract a true valley Zeeman splitting of (−7.9 ± 0.1)*μ*_B_*B* for all trion states, which may be measured only by consideration of both singlet and triplet trions, and attribute its deviation from the known atomic orbital contribution of ~ −4 *μ*_B_*B* to a strong Berry curvature associated magnetic moment unique to WSe_2_ trions. However, we observe this true trion valley Zeeman splitting to be masked by energetic recoil processes of the additional electron, which modify the measured trion energy shifts in low temperature magneto-PL studies and are likely to depend heavily on external factors such as doping level, which vary from sample to sample. This work removes the ambiguity surrounding the variation of trion valley Zeeman splittings reported in literature, by revealing the interplay between different trion complexes and external magnetic fields.

## Results

### Temperature dependent trion photoluminescence

**Fig. 2:**
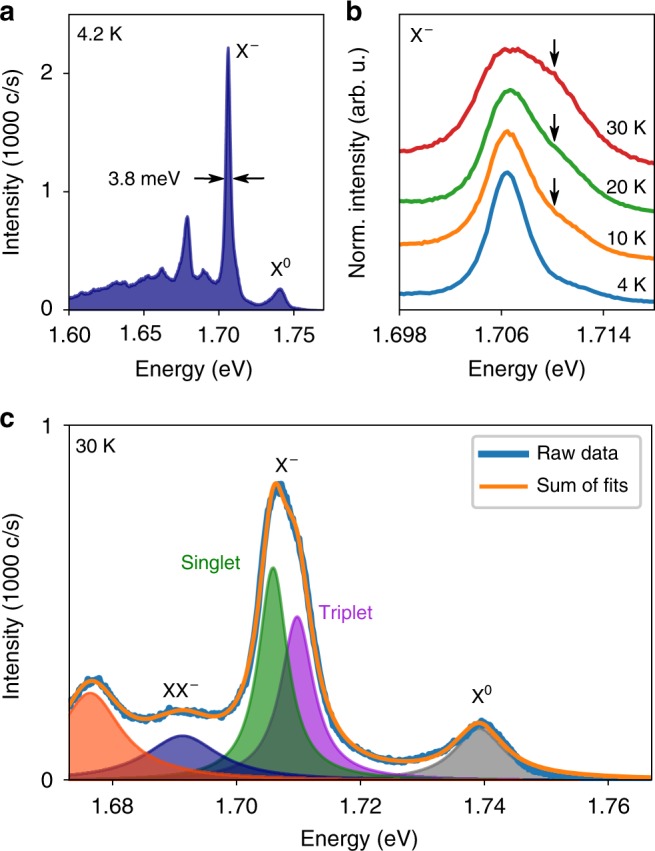
Trion photoluminescence in WSe_2_. **a** Photoluminescence (PL) spectrum from the sample under non-resonant laser excitation at 4.2 K. Peaks attributed to the neutral A-exciton (X^0^) and trion (X^−^) can be seen. The trion linewidth is 3.8 meV. **b** Temperature dependence of the trion photoluminescence from 4.2 to 30 K. Increasing thermal population of the higher energy triplet states can be inferred from the increasing spectral weight of the high energy shoulder of the trion peak, indicated by the black arrows. **c** PL spectrum from the sample at 30 K, fitted to five lorentzian peaks corresponding to various optically active states. The trion peak is well fitted to two peaks corresponding to the fine structure components, separated by ~4 meV. The navy blue peak may be the recently reported negatively charged biexciton, XX^−^, ~50 meV below X^0^

The sample used in this investigation consists of a monolayer of mechanically exfoliated WSe_2_, encapsulated on both sides by few-layer hexagonal boron nitride (hBN)^30^. Encapsulation in this manner is known to be responsible for narrow excitonic PL linewidths in TMD monolayers^31^, and is here responsible for a trion linewidth of 3.8 meV at 4.2 K, significantly narrower than the typical 10–20 meV values for bare WSe_2_^7^ and approaching the intrinsic homogeneous linewidth^32^. A PL spectrum from the sample at 4.2 K, excited by continuous-wave laser light at 1.946 eV, can be seen in Fig. 2a. Peaks corresponding to the neutral A exciton (X^0^) and negatively charged trion (X^−^) are visible, typical of WSe_2_^7^.

We observe in Fig. 2b, upon heating the sample from its base temperature of 4.2 K, an increasing spectral weight of the high energy shoulder of the trion peak, which we attribute to increasing thermal population of the triplet states, relative to the singlet states. Overall, the singlet emission becomes dimmer with increasing temperature, while the triplet emission becomes brighter (Supplementary Note 2). At 30 K, the triplet luminescence is of comparable intensity to the singlet. Figure 2c shows the region of interest of the PL spectrum at 30 K, fitted to five Lorentzian peaks, each corresponding to a different emissive state. The trion is accurately fitted by a doublet peak structure, indicating the presence of non-degenerate singlet and triplet trion emission. The singlet is 33 meV below X^0^, and the triplet is 29 meV below, meaning *δ*_ex_ = 4 meV. This value is smaller than previously reported values ≈6–7 meV^26^, with the difference likely due to influences of the dielectric environment on the relative singlet and triplet binding energies in our fully encapsulated sample, described by the Keldysh potential^33,34^. The fourth peak is 48 meV below X^0^, very close to the expected binding energy of the recently reported negatively charged biexciton^12–15^. For the remainder of this investigation, the sample was maintained at 30 K, where the comparable populations of singlet and triplet states allows the greatest insight into the magneto-optical response of the trion fine structure.

### Magneto-optical response of singlet and triplet trions

**Fig. 3:**
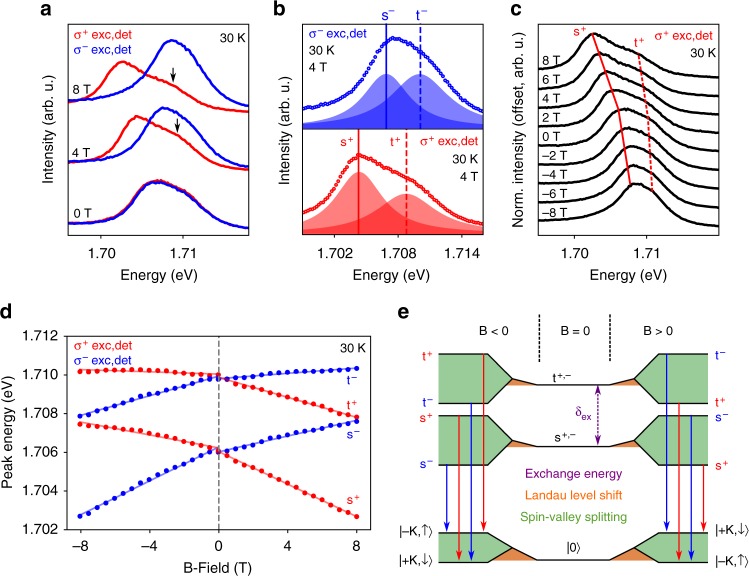
Photoluminescence of singlet and triplet trions under external magnetic fields. **a** Circular polarization resolved trion PL spectra at *B* = 0 T, 4 T, and 8 T, at 30 K. Oppositely polarized PL components shift spectrally away from each other with increasing B-field. The energy separation between trion fine structure components appears dependent on *B*-field, as evidenced by the growing separation between the main peak and the shoulder (indicated by black arrows) in *σ*^+^ polarization (red curve) with increasing field strength. The increasing symmetry and intensity of the emission in *σ*^−^ polarization (blue curve) suggests converging fine structure components with *B*. **b** The trion fine structure may be fitted to two Lorentzian peaks in each photon helicity, showing all four trion configurations at *B* = 4 T. Open circles are the CCD data. **c** B-field dependent trion PL in *σ*^+^ polarization. Solid and dashed red lines trace the approximate peak energies of the fitted Lorentzian functions for s^+^ and t^+^, respectively, shown in **b**. **d** Fitted peak energies of the fine structure components as a function of external *B*-field strength. Solid lines are linear fits to the data for either positive or negative field ranges. **e** Energy level diagram showing initial (one of the four trion varieties) and final (photon plus single electron in band c1) states of trion optical recombination, at positive and negative *B*-fields. The intervalley electron-hole exchange interaction creates the energy gap *δ*_ex_, lifting the degeneracy between singlet and triplet trions. When *B* ≠ 0, energy shifts arise from Landau level quantization of both trions and free electrons (orange areas), along with spin and valley associated magnetic moments (green areas). See main text for details. Energies are not to scale

At 30 K, and initially at zero external B-field, we observe in PL an asymmetric trion feature composed of an unresolved lower energy singlet peak and higher energy triplet peak, as can be seen in the 0 T trace of Fig. 3a. As depicted in Fig. 1, emission from t^+^ (s^+^) and t^−^ (s^−^) is at the same energy in the absence of an external B-field, and there is a few meV energy gap *δ*_ex_ between the triplet emission and singlet emission, arising from the intervalley exchange coupling.

Upon applying an external B-field perpendicular to the sample, up to *B* = 8 T, the *σ*^+^ and *σ*^−^ components of the emission shift spectrally away from one another, with *σ*^−^ emission shifting to higher energy, consistent with the valley Zeeman effect. However, as is clear from the 4 T and 8 T traces of Fig. 3a, there is an accompanying lineshape evolution of the trion feature with external field. It appears that when shifting to lower energy, the singlet and triplet increase their energy separation, as evidenced by the prominent shoulder appearing in the *σ*^+^ emission at *B* > 0, highlighted by black arrows. Conversely, when shifting to higher energy, the singlet and triplet peaks appear to reduce their energy separation, resulting in the overall brighter and narrower emission profile seen in the *σ*^−^ emission at *B* > 0.

By applying the same fitting procedure as used in Fig. 2c for zero-field PL, the photon energies of the four different trion states, i.e. s^+^, s^−^, t^+^, t^−^, may be extracted as a function of external magnetic field at 30 K. An example of the fitted fine structure is shown in Fig. 3b, at *B* = 4 T, where it is clear that four distinct peaks exist in total, corresponding to the four ground state trion varieties. Each of these fitted Lorentzian peaks is distinct from the other three by either photon energy, helicity, or both. As such, it is possible to isolate and trace the energy shift of each trion state independently over the external *B*-field range, as shown for s^+^ and t^+^ overlaid on the raw *σ*^+^ spectra in Fig. 3c. Figure 3d plots the fitted peak energies of all four trion varieties over the entire B-field range, from which two key observations can be made. Firstly, there is a much larger apparent valley splitting between singlet trions than between triplet trions, consistent with what may be inferred from the raw spectra in Fig. 3c. Secondly, there is a small redshift affecting all four trion states which appears to depend only on the magnitude of the B-field, and is independent of sign. As such, when *B* > 0, the *σ*^−^ trions appear to have lower rates of shift than their *σ*^+^ counterparts, in other words, t^−^ (s^−^) is less sensitive to the external field than t^+^ (s^+^). Conversely, when *B* < 0, t^+^ (s^+^) is less sensitive than t^−^ (s^−^), resulting in a striking change of gradient as each trion state crosses *B* = 0 T in Fig. 3d.

### Interpretation of trion photoluminescence peak shifts

To understand this behaviour, it is useful to consider the initial and final states of trion radiative recombination, under the influence of an external magnetic field in the Faraday geometry. The initial state consists of either a singlet trion, or a triplet trion at a raised energy *δ*_ex_. The additional electron present in each of these trion complexes ensures the initial state charge = −1, which quantizes the trion dispersion into Landau levels (LLs), with a cyclotron frequency $\omega _{{\mathrm{X}}^ - } = e|B|/m_{{\mathrm{X}}^ - }$ where *e* is the electron charge and $m_{{\mathrm{X}}^ - }$ the trion effective mass. Furthermore, the initial state is subject to energy shifts arising from the atomic orbital and Berry curvature associated magnetic moments inherent to monolayer WSe_2_, much like the neutral exciton^20–22,25^. The atomic orbitals constituting the valence band edge (band v1) have a magnetic moment of magnitude 2 *μ*_B_, which leads to an expected valley splitting of magnitude 4 *μ*_B_*B*. Any discrepancy from this value arises due to the Berry curvature, or so-called valley magnetic moment, which is associated with the valley pseudospin^20,21^. In analogy to the neutral exciton valley Zeeman effect, we can express the energy shift of the initial state trion as $\frac{1}{2}\tau _{\mathrm{z}}g_{\mathrm{z}}\mu _{\mathrm{B}}B$ where *τ*_z_ = ±1 for *σ*^±^ emission helicity, and *g*_z_ describes the cumulative effect of atomic orbital and Berry curvature magnetic moments.

From the initial state t^−^ or s^+^ (t^+^ or s^−^), the final state after trion recombination will be a photon and a single electron in the conduction band state |+K, ↓〉 (|−K, ↑〉). In each of these two final states, the electron experiences magnetic moments due to both the spin and valley pseudospin, which counteract one another. The cumulative spin-valley electron g-factor *g*_e_ in band c1 therefore depends on the relative strengths of these two opposing magnetic moments. The energy shift of an electron in band c1 may be expressed as $\frac{1}{2}\tau _{\mathrm{e}}g_{\mathrm{e}}\mu _{\mathrm{B}}B$ where *τ*_e_ = ±1 for the electron in the ±K valley, as a consequence of time reversal symmetry. The electron recoil effect described by *g*_e_ is trion-specific, and is responsible for the larger singlet-singlet splitting in Fig. 3d than triplet-triplet splitting.

In addition to the spin and valley energy shifts, the final state electron is also subject to LL quantization, however, the electron cyclotron energy *ω*_e_ will be much larger than $\omega _{{\mathrm{X}}^ - }$ thanks to the much smaller electron effective mass. Consequently, when a trion radiatively recombines, the additional energy of the electron LL relative to the trion LL is deducted from the photon energy. This leads to a global redshift of trion PL with increasing B-field magnitude, which may be quantified by an effective g-factor *g*_l_ as $\hbar \omega _{\mathrm{e}}(n_{\mathrm{e}} + 1) - \hbar \omega _{{\mathrm{X}}^ - }(n_{{\mathrm{X}}^ - } + 1) = g_{\mathrm{l}}\mu _{\mathrm{B}}|B|$, where *n*_e_ and $n_{{\mathrm{X}}^ - }$ are the LL index of the electron and trion, respectively. In Fig. 3d, *g*_l_ gives rise to the inequivalent rates of shift of each trion state between positive and negative *B*-field.

Overall, we define the change in emitted photon energy Δ*E*_*hν*_ as a function of the change in external magnetic field, Δ*B* as

1 $${\mathrm{\Delta }}E_{h\nu } = \frac{1}{2}(\tau _{\mathrm{z}}g_{\mathrm{z}} - \tau _{\mathrm{e}}g_{\mathrm{e}})\mu _{\mathrm{B}}{\mathrm{\Delta }}B - g_{\mathrm{l}}\mu _{\mathrm{B}}\left| {{\mathrm{\Delta }}B} \right|$$

where *g*_z_ may be viewed as the excitonic valley Zeeman g-factor of the trion, equal for all trion states, and *g*_e_ and *g*_l_ are modifications to the emitted photon energy arising purely from the recoil energy of the excess electron. The relative photon energies associated with Eq. (1) when *B* ≠ 0 are shown schematically in Fig. 3e. We note that in order to reproduce the data in Fig. 3d, the band c1 valley magnetic moment must have larger magnitude than the spin magnetic moment, such that under a positive external *B*-field, the state |+K, ↓〉 is at higher energy than |−K, ↑〉, considering that these two magnetic moments have opposite sign in c1^20^.

### Extraction of contributory g-factors

**Fig. 4:**
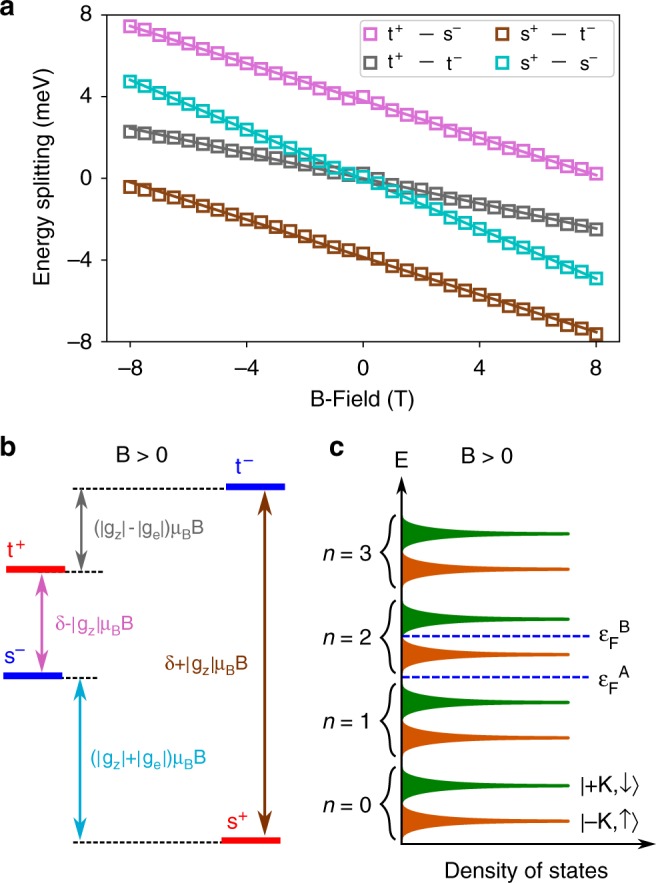
The valley Zeeman effect of trions in WSe_2_. **a** Photon energy separation as a function of external B-field between oppositely circularly polarized trion configurations, calculated from *E*(*σ*^+^) − *E*(*σ*^−^) in photoluminescence. Open squares are data points, while solid lines are linear fits used to extract gradients. **b** Schematic of the energy separations between photon energies from each trion state when B > 0. Solid red or blue lines specifically indicate photon energy in PL. The colour of text and arrows corresponds with the data in **a**. **c** Illustration of spin-valley polarized Landau levels in the band c1 when *B* > 0. The density of states is quantized into LLs separated by the cyclotron energy due to the *B*-field. These LLs then split into pairs due to the magnetic moment of the band c1. $\varepsilon _{\mathrm{F}}^{\mathrm{A}}$ and $\varepsilon _{\mathrm{F}}^{\mathrm{B}}$ indicate the Fermi energy in two different scenarios *A* and *B* described in the main text

In order to extract these various g-factors from the magneto-PL measurements, we consider the photon energy separations between trions of opposite PL polarization, in the convention *E*(*σ*^+^) − *E*(*σ*^−^), as plotted in Fig. 4a. Remarkably, despite the complexities of three distinct g-factors acting on four distinct trion states, the inherent symmetries in the system cause the energy splittings to become quite simplistic. Table 1 lists the measured gradients of each line in Fig. 4a, and the corresponding description calculated from Eq. (1). The associated energy separations when B > 0 are shown schematically in Fig. 4b. Table 1 reveals that *g*_z_ = −7.9 ± 0.1, corresponding to a trion valley Zeeman splitting of ≈−7.9 *μ*_B_*B*. This is approximately double the value expected from purely atomic orbital contributions in the valence band (−4 *μ*_B_*B*), implying a large Berry curvature associated magnetic moment of all trion states before recombination, present in the initial state but absent in the final state, in agreement with previous suggestions^21,25,29^. The opening of the energy gap *δ*_ex_ between oppositely circularly polarized dispersion minima (Fig. 1), absent for neutral excitons, transforms the trion into a massive Dirac particle, associated with a large Berry curvature Ω(**k**)^5,25^. The contribution to *g*_z_ from the Berry curvature may be expressed as $\frac{{m_{\mathrm{e}}}}{{2\hbar ^2}}\delta _{{\mathrm{ex}}}\Omega (\mathbf{k})$^21^ (Supplementary Note 1). Our data suggest that this contribution amounts to ~4 (as |*g*_z_| ≈ 8 and the atomic orbital contribution ~4), which yields a value of Ω(±*K*) ~ 10^4^ Å^2^. This is in excellent agreement with the predicted value when modelling the trion as a massive Dirac fermion^25^.

**Table 1:** Trion energy splitting values

Energy separation	Measured gradient	Corresponds to:
*E*(s^+^) − *E*(t^−^)	(−7.9 ± 0.1)*μ*_B_	*g*_z_ *μ*_B_
*E*(t^+^) − *E*(s^−^)	(−7.9 ± 0.1)*μ*_B_	*g*_z_ *μ*_B_
*E*(t^+^) − *E*(t^−^)	(−5.3 ± 0.1)*μ*_B_	(*g*_z_ + *g*_e_)*μ*_B_
*E*(s^+^) − *E*(s^−^)	(−10.5 ± 0.1)*μ*_B_	(*g*_z_ − *g*_e_)*μ*_B_

List of measured gradients extracted from data shown in Fig. 4a, and their representations in terms of *g*_z_ and *g*_e_ from Eq. (1), illustrated in Fig. 4b. The data suggest *g*_z_ = −7.9 ± 0.1 and *g*_e_ = 2.6 ± 0.1. See main text for details

From Table 1 we also extract *g*_e_ by inserting *g*_z_ = −7.9 ± 0.1 into (*g*_z_ ± *g*_e_)*μ*_B_. The result is a valley splitting of band c1 of *E*_+K,↓_ − *E*_−K,↑_ = *g*_e_*μ*_B_*B* = (2.6 ± 0.1)*μ*_B_*B*, corresponding to a single electron magnetic moment of ±(1.3 ± 0.1)*μ*_B_ in the ±K valley of band c1. This is in good agreement with the predicted c1 magnetic moment in a single particle picture, which is ~1.5 *μ*_B_^35^. This value is the sum of spin and valley magnetic moments, where the latter is a type of orbital magnetic moment arising from cyclic motion of electron Bloch states in momentum space, with opposite winding number between valleys^36^.

Inserting *g*_z _= −7.9 ± 0.1 and *g*_e_ = 2.6 ± 0.1 into Eq. (1) allows extraction of *g*_l_ by taking the gradients of the linear fits in Fig. 3d. Calculating *g*_l_ from each of the eight gradients (positive and negative *B*-field ranges for each of the four trion states) yields a mean *g*_l_ = 1.9 ± 0.1. Taking typical electron and trion effective masses from published density functional theory calculations yields a good agreement with the measured values of *g*_l_^37^ (Supplementary Note 1). Table 2 lists the three g-factors in Eq. (1) extracted in this study.

**Table 2:** Summary of extracted g-factors

g-factor	Extracted value	Physical origin
*g*_z_	−7.9 ± 0.1	Trion valley Zeeman splitting
*g*_e_	2.6 ± 0.1	Band c1 spin-valley splitting
*g*_l_	1.9 ± 0.1	Trion → electron LL energy

Summary of extracted g-factors in this study, corresponding to Eq. (1).

## Discussion

In this work, we reveal the complexities of trion magneto-PL, demonstrating that the trion energy splitting in PL does not reflect the underlying valley Zeeman splitting of the initial state. Instead, the process of trion radiative recombination itself modifies the emitted photon energy, via electron recoil to valley polarized conduction band states, having the effect of enhancing the singlet-singlet splitting, and diminishing the triplet-triplet splitting. On top of this asymmetry, we observe the action of a third process, also associated with the electron recoil, which we attribute to Landau level quantization of both initial and final states. Crucially, the end result is that each of the four ground state optically bright trion varieties experiences its own unique rate of shift when an external magnetic field is applied.

We arrive at the significant conclusion that any measurement of the trion valley splitting when treating it as a single resonance (without fine structure) cannot yield an accurate measurement of the true valley Zeeman effect, as the measured value will depend on the relative contributions of the four fine structure components, each of which have different rates of shift, as shown in Fig. 3d, alongside variable relative PL intensities, as shown in Figs. 2b and 3b. To our knowledge, all as-yet published reports of the trion valley splitting in WSe_2_ treat the trion as a single spectral peak, and so it cannot be excluded that the varying contributions to the overall trion PL from the four underlying states may have influenced the measured g-factors in an uncontrolled manner. To compound the problem, typical exfoliated WSe_2_ monolayers exhibit trion linewidths much broader than the exchange energy separation of 4–7 meV, making it impossible to isolate the fine structure components in all but the highest quality samples. To demonstrate how neglecting the fine structure can lead to extraction of erroneous g-factors, we measure the valley splitting of the total trion emission (the spectral feature encompassing both singlet and triplet PL) at 4 K and 30 K, taking the energy of the brightest CCD pixel as the peak energy (Supplementary Note 3). We extract values of *E*(*σ*^+^) − *E*(*σ*^−^) = (−12.4 ± 0.1)*μ*_B_*B* and (−12.8 ± 0.2)*μ*_B_*B* at 4 K and 30 K, respectively, neither of which have any genuine physical meaning, both being influenced by shifting substructure. Only by simultaneous consideration of both singlet and triplet trions is it possible to extract the strength of the valley splitting of the initial states, *g*_z_.

Next, we consider *g*_l_, which describes the asymmetry between rates of shift of the same trion state at positive and negative *B*-fields. Landau level spectroscopy performed on monolayer WSe_2_ has revealed that many-body interactions are quite weak in the band c1, ensuring that a single particle picture remains valid to describe the dynamics of the excess electron upon recoil^35^. We propose two regimes whereby *g*_l_ takes either the same value regardless of excess electron valley index, or inequivalent values, depending on the Fermi level of the sample. These are illustrated in Fig. 4c. The cyclotron energy in the band c1 has been shown to be larger than the valley splitting, leading to a staggered energy ladder of successive LLs when *B* > 0^35,38^. In the scenario A, the next available empty states which the excess electron can occupy upon trion recombination are the *n* = 2 LLs in both valleys. Therefore, *g*_l_ should be equal regardless of excess electron valley index. However, in the scenario B, the *n* = 2 LL is completely filled in the −K valley, but empty in +K. As such, the excess electron must occupy a LL of different *n* in opposite valleys. This would induce an additional PL redshift, amounting to ℏ*ω*_e_, from trion states with the excess electron in the −K valley. This picture is simplistic and neglects LL broadening^39^, or the kinetic energy involved in trion recombination, which are all likely to depend on temperature. However, it demonstrates how *g*_l_ can take inequivalent values for different trion states, depending on the arbitrary Fermi level in a given sample, offering another contribution to the wide ranging trion valley splittings reported in literature. While in our theoretical model we attribute the observed redshift to Landau level effects in the initial and final states of trion recombination, we note that there may be an increase in trion binding energy with increasing magnetic field strength, which would also contribute a slight redshift to the observed PL emission with increasing |*B*|^40^. Both of these processes may contribute to the redshift component which we measure and quantify with *g*_l_. To fully separate these possible contributions to *g*_l_, experiments with electron density controlled samples in high magnetic fields are required, to enter the regime of low LL filling factor, and significant binding energy modification.

In conclusion, we exploit the unique valley symmetries of WSe_2_ trion fine structure to optically measure all effective g-factors arising from valley Zeeman and electron recoil processes, which reveals that the valley Zeeman framework reported for neutral excitons is insufficient to describe the magneto-optical response of trions in WSe_2_. From purely optical measurements, we extract the single electron magnetic moment in the band c1, shedding new light on the magnetic response of dark excitons in this material, the properties of which are highly elusive on account of their spin forbidden optical transition^41^. Recently, owing to improved fabrication methods and encapsulation in pristine hBN, the overall quality of monolayer WSe_2_ samples has improved remarkably, amply demonstrated by the recent observation of negatively charged biexcitons. We envisage future magneto-optical studies of this and other excitonic species, building on this work, and leading towards a full and complete understanding of the electronic and optical properties of monolayer semiconductors. The results presented here gain critical insight into the magneto-photoluminescence of trion fine structure in monolayer WSe_2_, information which will be crucial in future research involving the spin and valley dynamics of monolayer TMDs and their applications in valleytronics.

## Methods

### Low temperature magneto-optical spectroscopy

Low temperature magneto-photoluminescence spectroscopy was performed by mounting the sample in a liquid helium bath cryostat containing a sample heating element and superconducting magnet coil. Non-resonant continuous-wave excitation at 1.946 eV in either *σ*^+^ or *σ*^−^ polarization was used, along with helicity selective circularly polarized detection, achieved by incorporating waveplates into the optical path before and after the sample. The PL was guided to a spectrometer and high sensitivity CCD.

### Sample fabrication

The hBN/WSe_2_/hBN stack was fabricated as follows. Firstly, bulk hBN crystals were mechanically exfoliated onto a polymer double layer commonly used for dry-transfer methods^42^. The WSe_2_ single-layer flake was then picked up from a separate Si/SiO_2_ substrate using the hBN crystal on the poly(methyl methacrylate) (PMMA) membrane. This pick-up method was repeated to lift another thin hBN flake from a second Si/SiO_2_ substrate. The WSe_2_ monolayer crystal is then fully protected from subsequent environmental degradation. The pick-up transfer was conducted with the target substrate held at *T* = 60 °C. The whole stack along with the PMMA membrane was then lowered onto a dielectric distributed Bragg reflector (DBR) substrate. The substrate consists of alternating layers of Ta_2_O_5_ and SiO_2_ of ~100 nm thickness, with SiO_2_ as the top layer. The PMMA membrane along with the heterostructure stack was heated to 130 °C to soften the PMMA. Subsequent electron beam lithography and metallization was carried out to mechanically clamp as well as aid the locating of the heterostructure on the substrate.

## Supplementary information

Supplementary Information

## Data Availability

All relevant data is available from the authors upon request.
