# Supplementary material for: The valley Zeeman effect in inter- and intra-valley trions in monolayer WSe2
Source: Nat Commun. 2019 May 27;10:2330. doi: 10.1038/s41467-019-10228-7 (PMC6536528; doi:10.1038/s41467-019-10228-7)
Supplement: Supplementary file 1 — Supplementary Information [file 41467_2019_10228_MOESM1_ESM.pdf]

Supplementary Information: The valley Zeeman effect in inter- and intra-valley trions in monolayer WSe_2

T. P. Lyons *et al.*

Supplementary Note 1: A model for the trion valley Zeeman effect in monolayer WSe_2

A negatively charged trion in a WSe_2 monolayer with a perpendicular magnetic field may be described by the following effective Hamiltonian

$$\mathcal{H} = H_T + H_{\text{Int}} + H_{SO} + H_Z \tag{1}$$

where H_T describes the kinetic energy of all three constituent particles of the trion in a magnetic field, H_{Int} describes the coulomb interactions, H_{SO} the spin orbit splitting and H_Z describes the Zeeman energies as follows

$$H_T = \sum_{i=1}^{N=3} \left(-\frac{\hbar^2}{2m_i} \boldsymbol{\nabla}_i + \frac{e}{\hbar} \mathbf{A}_i \right) \tag{2a}$$

$$H_{\text{Int}} = \sum_{i<j}^{N=3} V_{ij}(|\mathbf{r_i} - \mathbf{r_j}|) \tag{2b}$$

$$H_{SO} = \tau_1 s_1 \Delta_{vb} + \sum_{i=2}^{N=3} \tau_i s_i \Delta_{cb} \tag{2c}$$

$$H_Z = \frac{1}{2}(\tau_1 g_{vl}^{vb} + s_1 g_s^{vb} + \sum_{i=2}^{N=3} [\tau_i g_{vl}^{cb} + s_i g_s^{cb}]) \mu_B B_Z. \tag{2d}$$

Here $i = 1$ denotes the hole of the trion while $i = 2, 3$ denotes the bound and excess electron respectively. The effective masses and vector potential in the perpendicular magnetic field of the ith constituent particle is given by m_i and $\mathbf{A}_i$ respectively. $V_{ij}(r)$ describes the interaction potential between charged particles, which in a TMD monolayer is best described with the Keldysh potential[1] given as follows

$$V_{ij}(r) = \frac{e_1 e_2 \pi}{2r_0} \left[H_0 \left(\frac{\kappa r}{r_0} \right) - Y_0 \left(\frac{\kappa r}{r_0} \right) \right]. \tag{3}$$

Here r_0 is the screening length of the TMD, κ is the average dielectric constant of the surrounding environment (in this case just the dielectric constant of hBN), H_0 is the 0th Struve function and Y_0 is the 0th Bessel Y function. Lastly, $g_{vl}^{vb/cb}$ is the valley g-factor in the valence/conduction band and $g_s^{vb/cb}$ is the spin g-factor in the valence/conduction band while $\tau_i = \pm 1$ and $s_i = \pm 1$ give the associated quantum number of the spin and valley pseudospin of the ith particle (1 corresponding to the K valley or $\uparrow$ spin and -1 the K' valley or $\downarrow$ spin).

The full expression for the change in emitted photon energy due to negative trion recombination in a magnetic field is

$$\Delta E_{h\nu} = \Delta E_Z - \Delta E_{\text{R}} + \Delta E_{\text{D}} \tag{4}$$

where ΔE_Z is the Zeeman shift of the electron-hole pair within the trion, ΔE_{R} is the excess electron recoil shift and ΔE_{D} is the diamagnetic shift of the trion. The diamagnetic shift would induce a nonlinear shift in the observed trion lines with magnetic field. However, it is known from previous works[2, 3] that this nonlinearity is not expected to become relevant until field strengths of $> 15\,\text{T}$ are reached. As such, in this work, ΔE_{D} is not relevant.

Unlike exciton recombination, trion recombination cannot be a zero momentum process, as some momentum will be imparted to the excess electron as recoil, detracting from the observed photon energy. The excess electron recoil is dependent on both the temperature of the sample[4] and the Fermi level[5], both of which are kept constant within this experiment. The effect of a perpendicular magnetic field on the excess electron

recoil cost may be described by the difference in the trion and free electron first Landau level. Therefore the change in energy of the excess electron after trion recombination in a magnetic field is

$$\Delta E_e^\tau = \frac{1}{2}\hbar\omega_c\left(1 - \frac{m_3}{m_T}\right) + \frac{1}{2}\tau_e g_e \mu_B B_Z \tag{5}$$

where $\omega_c = eB_Z/m_3$ is the cyclotron frequency, τ_e is the valley index of the excess electron, $m_T = \sum_{i=1}^{N=3} m_i$ is the effective mass of the trion and g_e is the bare out of plane g-factor of an electron in WSe_2. As the recoil energy cost is equal in the two valleys and this entire expression is linear in magnetic field, an effective valley dependent g-factor model can be used

$$\Delta E_e^\tau = \frac{1}{2}\tau_e g_\tau \mu_B B_Z. \tag{6}$$

Here g_τ is the valley dependent g-factors of the excess electron given in the model as

$$g_\tau = g_e + \tau_e g_l \tag{7}$$

where (5) and (6) may be solved to give a Landau level associated g-factor of

$$g_l = \frac{2m_e m_X}{m_3 m_T} \tag{8}$$

where m_e is the bare electron mass and $m_X = \sum_{i=1}^{N=2} m_i$ is the exciton effective mass. Note the exciton mass here comes from the simplification of the Landau level component of (5) as we have assumed that the quasiparticle effective masses are linear combinations of the effective masses of the constituent particles. This accounts for the additional asymmetries of the observed photoluminescence energy gradients observed and detailed in Fig. 3d, but cancels out in the calculations of energy separations between different trion states, as shown in Table I of the main text, assuming g_l is equal between trion states. Fig. 4c of the main text demonstrates how g_l may take different values in different samples depending on the arbitrary Fermi level. Assuming a 2-dimensional electron gas, the electron density N_e at which g_l becomes uneven between the two valleys may be expressed as

$$\frac{eB_Z}{\pi\hbar}\left[\frac{m_e}{m_3}\left(\frac{1}{2}+n\right) - \frac{1}{4}g_e\right] < N_e < \frac{eB_Z}{\pi\hbar}\left[\frac{m_e}{m_3}\left(\frac{1}{2}+n\right) + \frac{1}{4}g_e\right] \tag{9}$$

where n is the index of the lowest accessible LL. At an external B-field strength of 10 T, an electron density of $2.8 \times 10^{12} m^{-2}$ will freeze out the zeroth LL in one valley only, breaking the symmetry of g_l. Using reasonable values of the effective masses for WSe_2 from Density Functional Theory (DFT) ($m_1 = -0.36m_e$ and $m_2 = m_3 = 0.29m_e$[6]) a $g_l = 2.2$ close to the measured value (~ 1.9) is found.

Considering the spin-valley combinations of the four bright trions measured in this work, two triplet states $t^\pm$ and two singlet states $s^\pm$ addressed by $\sigma^\pm$ circularly polarised light, the Zeeman splitting may be reformulated as

$$H_Z = g_{t/s}^\pm \mu_B B_Z = \frac{1}{2}(\tau_z g_z - \tau_e g_e - 2g_l)\mu_B B_Z. \tag{10}$$

Here, $g_{t/s}^\pm$ is the g-factor of a specific variant of the bright trion measured, which in turn is composed of the exciton g-factor g_z (given by the particles that will radiatively recombine) and the excess electron g-factor

g_e, both with associated valley indices τ_z and τ_e respectively. As such, the total valley g-factors of each of the measured trions are as follows

$$g_s^+ = \frac{1}{2}(g_z - g_e - 2g_l) \tag{11a}$$

$$g_s^- = -\frac{1}{2}(g_z - g_e + 2g_l) \tag{11b}$$

$$g_t^+ = \frac{1}{2}(g_z + g_e - 2g_l) \tag{11c}$$

$$g_t^- = -\frac{1}{2}(g_z + g_e + 2g_l). \tag{11d}$$

From this, the results shown in Table I. in the main text can be simply recovered

$$g_s^+ - g_s^- = g_z - g_e \tag{12a}$$

$$g_t^+ - g_t^- = g_z + g_e \tag{12b}$$

$$g_t^+ - g_s^- = g_z \tag{12c}$$

$$g_s^+ - g_t^- = g_z. \tag{12d}$$

Lastly, as is mentioned in the main text, there is a sizeable difference between what is measured as the valley Zeeman g-factor of the trion g_z and the expected contribution of the atomic orbital associated magnetic moment. This is most likely due to a significant shift of the experienced berry curvature $\Omega(\mathbf{k})$ and exchange energy of the trion[7] which contributes to the valley g-factor[8]. This additional contribution is part of the measured g_z from this experiment. It is known that there is an associated magnetic moment μ with the trion exchange energy

$$\mu(\mathbf{k}) = \frac{e}{2\hbar}\delta_{ex}\Omega(\mathbf{k}) \tag{13}$$

where δ_{ex} is the zero field exchange energy splitting between the singlet and triplet trion configurations which is $\delta_{ex} \sim 4\,\text{meV}$. The g factor associated with this magnetic moment is

$$g_\Omega = \frac{m_e}{2\hbar^2}\delta_{ex}\Omega(\mathbf{k}) \tag{14}$$

The data presented in the main text suggests g_Ω is of magnitude ~ 4, which from (13) suggests that $\Omega(\pm K) \sim 10^4$ Å at the K (K') valleys, in agreement with the expected value when modelling the trion with a massive Dirac Hamiltonian[7].

Supplementary Note 2: Temperature, B-field and laser power dependent photoluminescence spectra

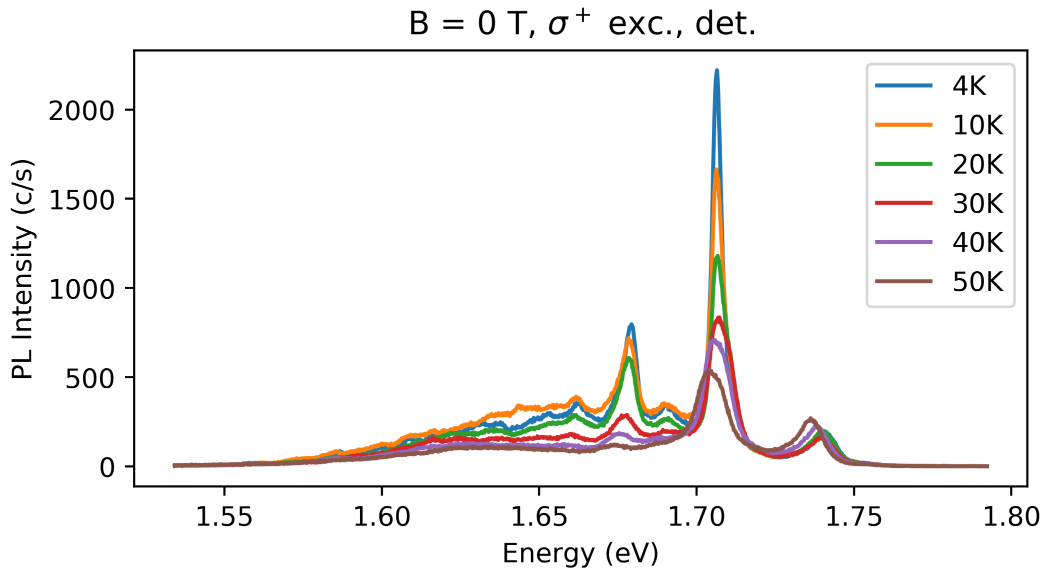

Supplementary Figure 1. PL spectra over the entire energy range from 4 K to 50 K at B = 0 T.

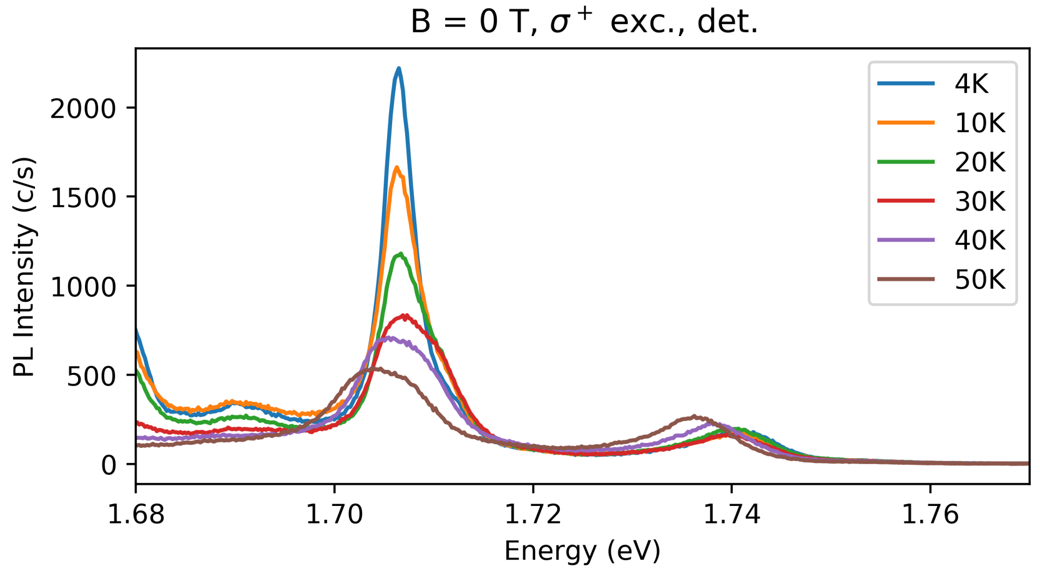

Supplementary Figure 2. PL spectra over the energy range covering X^0 and X^- from 4 K to 50 K at B = 0 T. The growth of the triplet trion is evident at 10, 20 and 30 K, while at 40 and 50 K the emission becomes broader and redshifts.

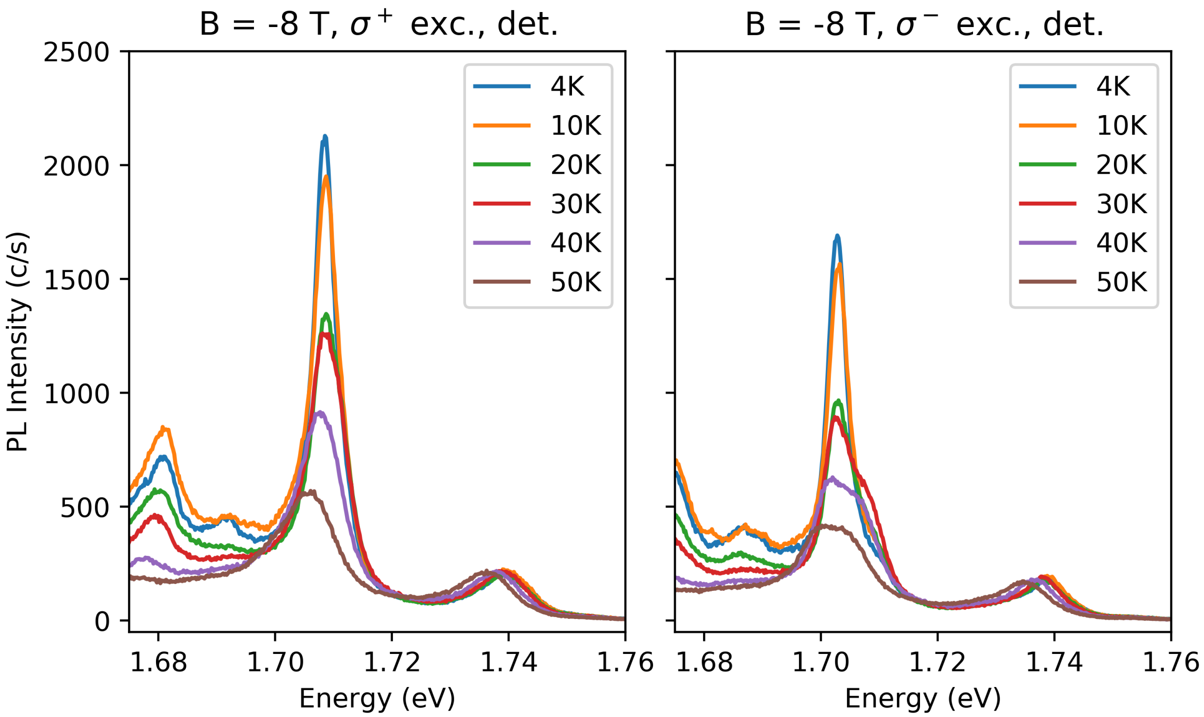

Supplementary Figure 3. PL spectra over the energy range covering X^0 and X^- from 4 K to 50 K at B = -8 T in both polarizations.

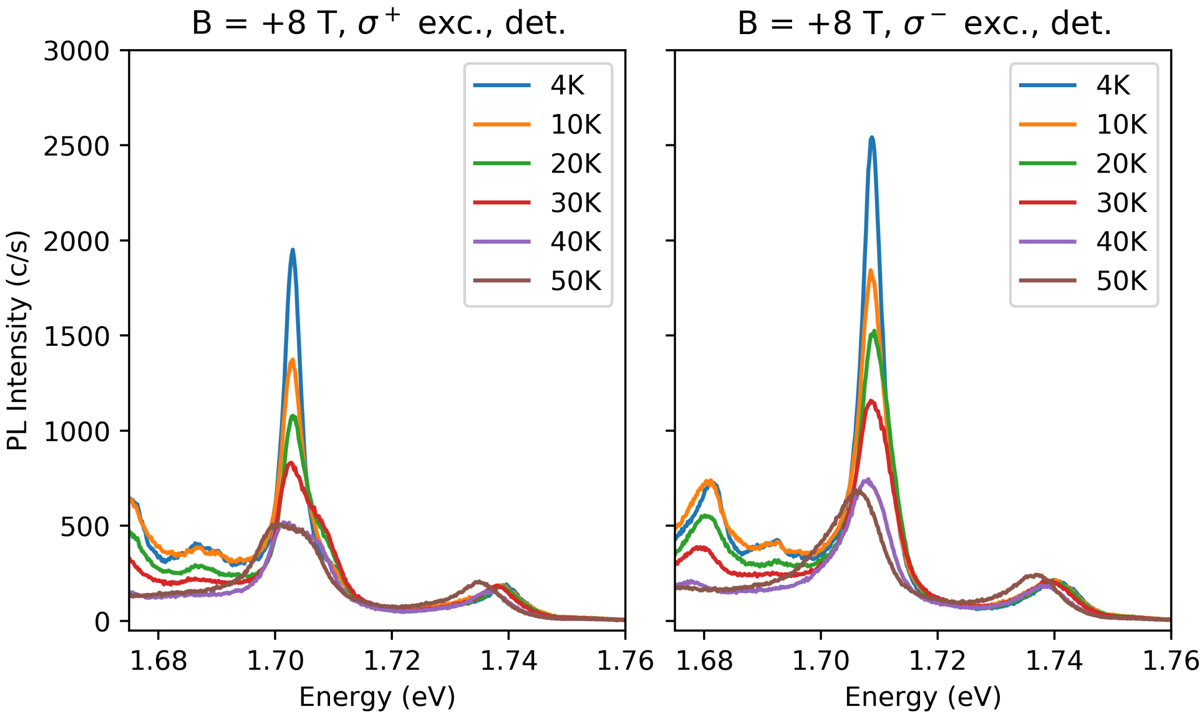

Supplementary Figure 4. PL spectra over the energy range covering X^0 and X^- from 4 K to 50 K at B = +8 T in both polarizations.

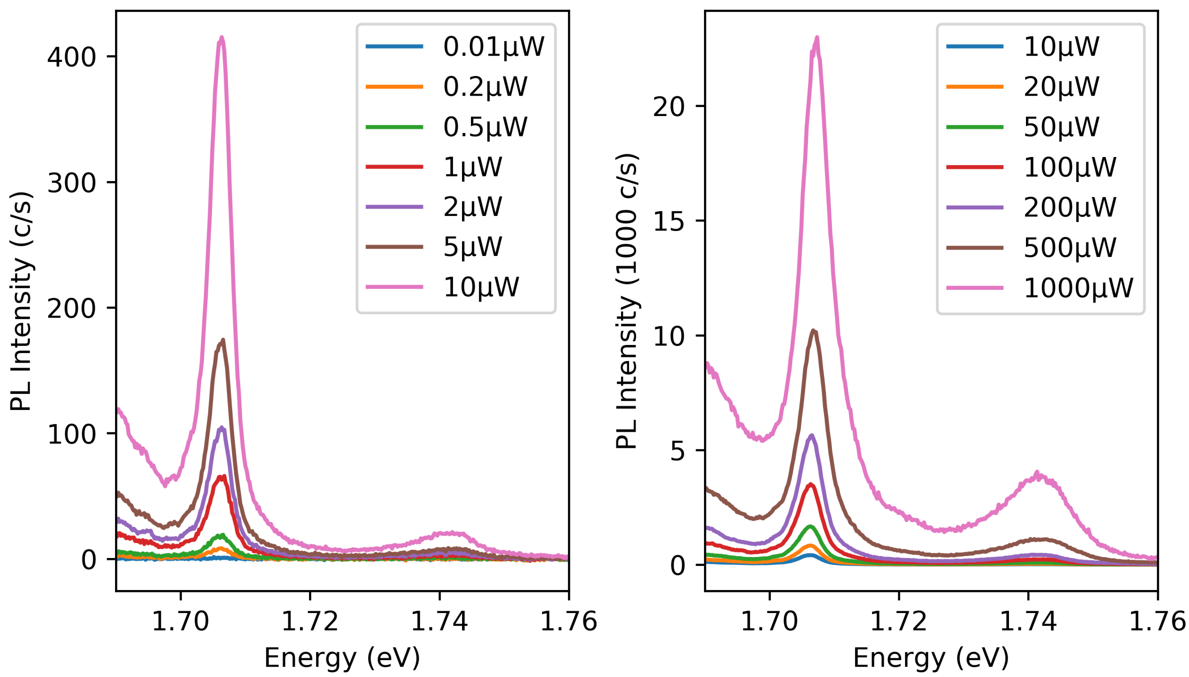

Supplementary Figure 5. PL spectra over the energy range covering X^0 and X^- at various laser excitation powers and B = 0 T.

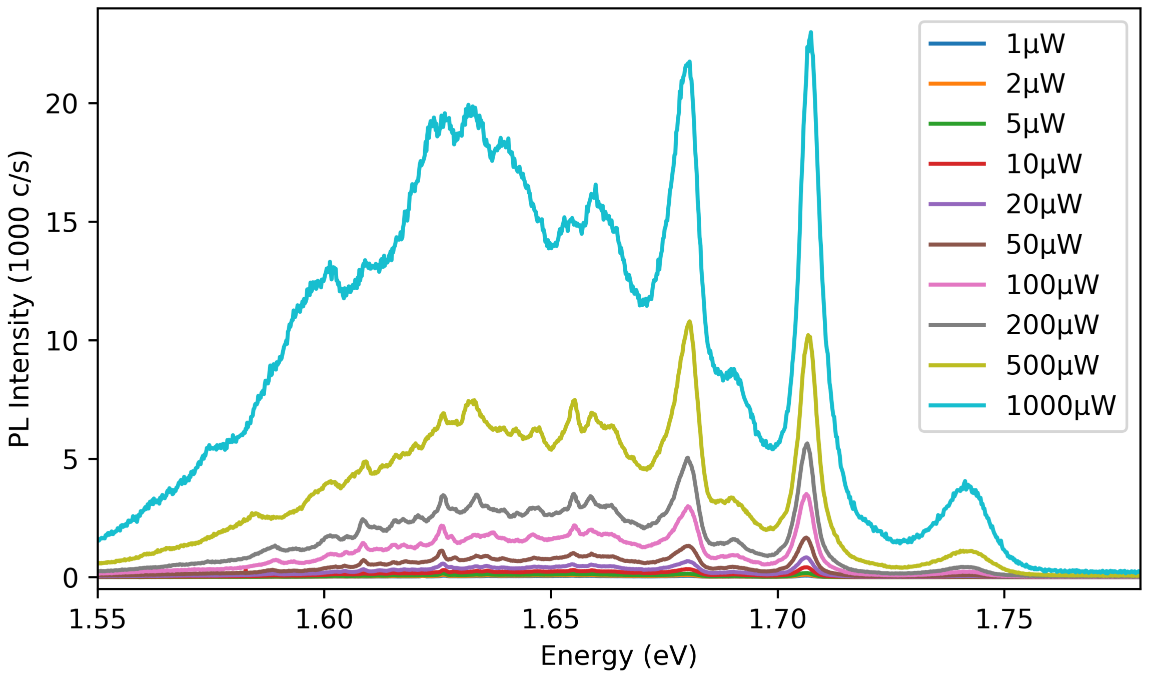

Supplementary Figure 6. PL spectra over the full energy range at various laser excitation powers and B = 0 T.

Supplementary Note 3: Comparison of 4 K and 30 K PL valley splitting

The main text demonstrates how singlet and triplet trions have unique rates of shift in PL when an external magnetic field is applied. This is proposed to be the underlying cause of the variation in trion g-factors reported in literature: in studies where the trion PL linewidth exceeds the exchange energy separation between fine structure components (as in all but the highest quality samples employing hBN encapsulation), the inequivalent contributions from unresolved singlet and triplet substructure cannot be precisely accounted for, leading to inaccurate measurement of the g-factor of the trion. To demonstrate this, we measure the valley splitting of the *total* trion emission (the PL peak comprising unresolved fine structure) at 4 K and 30 K, using peak energies extracted by two different methods. The "centre of mass" method is calculated as $(\sum(E \times I)_n)/(\sum I_n)$, where E and I are the energy and intensity of the nth pixel, whereas the "peak intensity" is the energy of the pixel with the most counts per second. The results are shown in the figure and table below, and reveal two key conclusions. Firstly, the centre of mass and peak intensity do not agree with each other at either temperature. This strongly indicates the presence of peak substructure which is not negligible. Secondly, none of these g-factors correspond to the values presented in the main text, accurately extracted using multiple peak fitting. This comparison demonstrates that when the trion fine structure is neglected, the uncontrolled influences on the overall PL line shifts can lead to extraction of valley Zeeman splittings ranging from $\sim -5\mu_B B$ to $\sim -13\mu_B B$, all from the same sample, and corresponding quite closely to the range of trion g-factors already reported in literature.

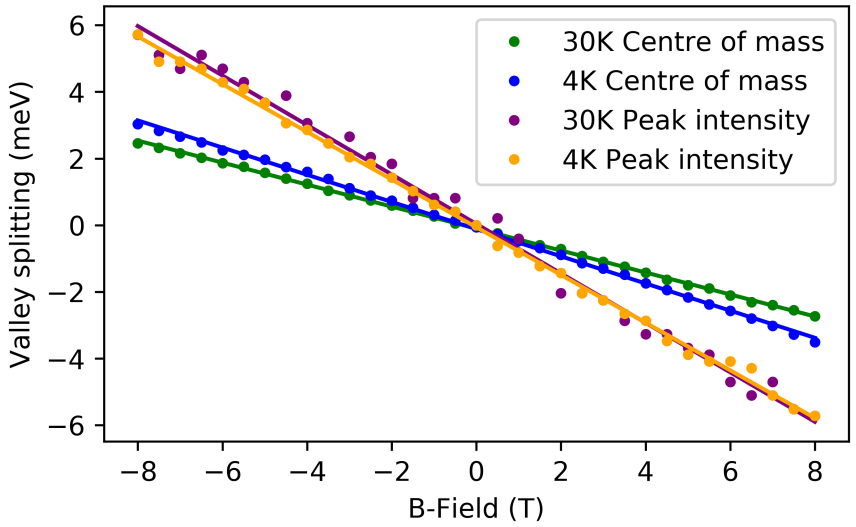

Supplementary Figure 7. Comparison of trion PL valley splitting $E(\sigma^+) - E(\sigma^-)$ measured by two different methods, both without taking the trion fine structure into account.

Temperature	Peak analysis method	Measured overall g-factor
4 K	Peak intensity	-12.8 ± 0.2
30 K	Peak intensity	-12.4 ± 0.1
4 K	Centre of mass	-7.04 ± 0.04
30 K	Centre of mass	-5.69 ± 0.02

Supplementary Table I. Summary of extracted trion PL g-factors ($E(\sigma^+) - E(\sigma^-) = g\mu_B B$) when the trion fine structure is not taken into account.

Supplementary References

[1] Courtade, E., *et al.* Charged excitons in monolayer WSe_2: experiment and theory. *Phys. Rev. B* **96,** 085302 (2017).

[2] Plechinger, G., *et al.* Excitonic valley effects in monolayer WS_2 under high magnetic fields. *Nano Lett.* **16(12),** 7899-7904 (2016).

[3] Stier, A. V., McCreary, K. M., Jonker, B. T., Kono, J., Crooker, S. A. Exciton diamagnetic shifts and valley Zeeman effects in monolayer WS_2 and MoS_2 to 65 Tesla. *Nature Comm.* **7,** 10643 (2016).

[4] Christopher, J. W., Goldberg, B. B., Swan, A. K. Long tailed trions in monolayer MoS_2: temperature dependent asymmetry and resulting red-shift of trion photoluminescence spectra. *Scientific Reports* **7,** 14062 (2017).

[5] Zhang, Q., *et al.* Recoil effect and photoemission splitting of trions in monolayer MoS_2. *ACS Nano.* **11(11),** 10808-10815 (2017).

[6] Kormányos, A., *et al.* k.p theory for two-dimensional transition metal dichalcogenide semiconductors. *2D Materials* **2,** 022001 (2015).

[7] Yu, H., Liu, G.-B., Gong, P., Xu, X., Yao, W. Dirac cones and Dirac saddle points of bright excitons in monolayer transition metal dichalcogenides. *Nature Comm.* **5,** 3876 (2014).

[8] Srivastava, A., Sidler, M., Allain, A. V., Lembke, D. S., Kis, A., Imamoğlu, A. Valley Zeeman effect in elementary optical excitations of monolayer WSe_2. *Nature Phys.* **11,** 141-147 (2015).
